# Transplant outcomes in positive complement-dependent cytotoxicity- versus flow cytometry-crossmatch kidney transplant recipients after successful desensitization: a retrospective study

**DOI:** 10.1186/s12882-019-1625-2

**Published:** 2019-12-09

**Authors:** Deok Gie Kim, Juhan Lee, Younhee Park, Myoung Soo Kim, Hyeon Joo Jeong, Soon Il Kim, Yu Seun Kim, Beom Seok Kim, Kyu Ha Huh

**Affiliations:** 1 Department of Surgery, Yonsei University Wonju College of Medicine, Wonju, Republic of Korea; 2 Department of Surgery, Yonsei University College of Medicine, Seoul, Republic of Korea; 3 Department of Laboratory Medicine, Yonsei University College of Medicine, Seoul, Republic of Korea; 4 The Research Institute for Transplantation, Yonsei University College of Medicine, Seoul, Republic of Korea; 5 Department of Pathology, Yonsei University College of Medicine, Seoul, Republic of Korea; 6 Department of Internal Medicine, Yonsei University College of Medicine, Seoul, Republic of Korea

**Keywords:** Kidney transplantation, Positive crossmatch, Donor-specific antibody, Desensitization

## Abstract

**Background:**

Despite the obvious survival benefit compared to that among waitlist patients, outcomes of positive crossmatch kidney transplantation (KT) are generally inferior to those of human leukocyte antigen (HLA)-compatible KT. This study aimed to compare the outcomes of positive complement-dependent cytotoxicity (CDC) crossmatch (CDC + FC+) and positive flow cytometric crossmatch (CDC-FC+) with those of HLA-compatible KT (CDC-FC-) after successful desensitization.

**Methods:**

We retrospectively analyzed 330 eligible patients who underwent KTs between June 2011 and August 2017: CDC-FC- (*n* = 274), CDC-FC+ (*n* = 39), and CDC + FC+ (*n* = 17). Desensitization protocol targeting donor-specific antibody (DSA) involved plasmapheresis, intravenous immunoglobulin (IVIG), and rituximab with/without bortezomib for positive-crossmatch KT.

**Results:**

Death-censored graft survival and patient survival were not different among the three groups. The median estimated glomerular filtration rate was significantly lower in the CDC + FC+ group than in the compatible group at 6 months (*P* < 0.001) and 2 years (*P* = 0.020). Biopsy-proven rejection within 1 year of CDC-FC-, CDC-FC+, and CDC + FC+ were 15.3, 28.2, and 47.0%, respectively. Urinary tract infections (*P* < 0.001), *Pneumocystis jirovecii* pneumonia (*P* < 0.001), and cytomegalovirus viremia (*P* < 0.001) were more frequent in CDC-FC+ and CDC + FC+ than in CDC-FC-.

**Conclusions:**

This study showed that similar graft and patient survival was achieved in CDC-FC+ and CDC + FC+ KT compared with CDC-FC- through DSA-targeted desensitization despite the higher incidence of rejection and infection than that in compatible KT.

## Background

Patients undergoing positive-crossmatch kidney transplantation (KT) from living donors have the advantage of survival compared to highly sensitized patients on waitlists [1]. However, there is inevitable immunologic risk despite rigorous pre- or posttransplant immunosuppression during desensitization. Several studies have reported a higher graft failure and mortality after positive-crossmatch KT than after compatible KT [2–4]; hence, only certain centers perform positive-crossmatch KT in the United States [5].

Methods based on complement-dependent cytotoxicity (CDC) crossmatch for identifying and characterizing anti-HLA antibody, such as the single-antigen assay and the C1q assay, have recently been developed and used clinically [6, 7]. Since the introduction of more sensitive methods such as flow cytometry (FC) crossmatch and the single antigen assay (SAA) using Luminex technology, the importance of quantifying antigen strength has been emphasized [6, 8–12]. Furthermore, baseline donor-specific antibody (DSA) titers are associated with the outcome of positive-crossmatch KT [13]. However, most studies on positive-crossmatch KT so far did not examine preoperative DSA titers, but rather involved retrospective examinations, thus not indicating DSA to be an appropriate target for successful desensitization.

A recent study reported a similar graft and patient survival rate between positive-crossmatch living-donor KT (LDKT) and compatible KT upon desensitization with a risk-stratified protocol [14]; however, the study involved a small cohort undergoing CDC-positive KT. Hence, this study aimed to evaluate post-transplant outcomes of CDC-positive (CDC + FC+) and FC-positive (CDC-FC+) KT using a DSA-targeted desensitization protocol and compare these outcomes with those of compatible KT (CDC-FC-).

## Methods

### Study population

**Fig. 1:**
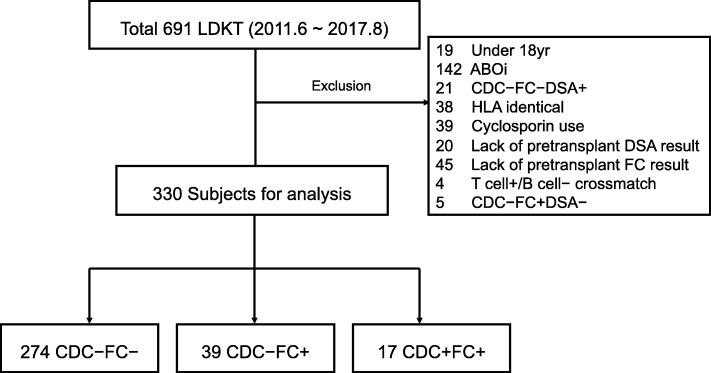
Study population. CDC, complement dependent cytotoxicity; DSA, donor specific antibody; FC, flow cytometry; HLA, human leukocyte antigen; LDKT, living donor kidney transplantation

We retrospectively assessed 691 consecutive living donor KT recipients at Severance hospital in Seoul, Korea from June 2011, when Luminex-based SAA was initiated, to August 2017. Patients under 18 years of age, those receiving kidney transplants from HLA-identical siblings, ABO incompatible KT, those taking cyclosporin primarily as an immunosuppressant, and those lacking findings of pretransplant DSA or FC were excluded. Cases of negative crossmatch with DSA (*n* = 21) were excluded in accordance with the recent report on non-inferior graft survival [15]. Furthermore, patients presenting positive T-cell but negative B-cell crossmatches and positive FC without DSA were excluded owing to potential technical errors. Finally, 330 eligible patients were divided into three groups; CDC-FC-, CDC-FC+, and CDC + FC+ (Fig. 1).

### Crossmatch and detection of DSA

Throughout the study, CDC and FC crossmatch and SAA were screened for all KT recipients with their potential living donors and repeated 1 or 2 days before transplantation. T-cell CDC was performed with anti-human globulin test and B-cell CDC was performed by warm-method. The results were recorded as the maximum titer presenting more than 11% of cell lysis. FC crossmatch using FACS Canto II (Beckton Dickinson, San Jose, CA, USA) was considered positive when the median fluorescence intensity (MFI) ratio was greater than 2.0. SAA was performed using two reagents during the study period: 1. Lifecodes LSA class I and class II (Gen-Probe Transplant Diagnostics, Inc., Stamford, CT, USA) from Jun 2011 to Dec 2014; 2. LABScreen Single Antigen class I and class II (One Lambda, Canoga Park, CA, USA) from Jan 2015 to Aug 2017. Antibodies against donor HLA-A, B, DR, and DQ were characterized, and the strength of each DSA was determined at the maximum MFI value. An MFI of > 1000 was considered positive. We assessed DSA in all recipients before KT. After transplantation, DSA was checked only when rejection was suspected until September 2015 due to cost issue. Since then, SAA for the detection of DSA has been covered by national insurance in our country and we checked DSA at least once a year regardless of acute rejection. For CDC-FC+ and CDC + FC+ groups, routine DSA assessment was performed more frequently according to pretransplant antigen strength and clinical course, at least every 3 months during 1 posttransplant year. C1q binding ability of anti-HLA DSA was examined in accordance with a previously reported method (One Lambda) [7].

### Desensitization protocols and immunosuppression

Pretransplant desensitization for positive-crossmatch KT at our institution involves plasmapheresis (PP)/low dose intravenous immunoglobulin (IVIG, 100 mg/kg per cycle) and rituximab (375 mg/m^2^) (Additional file 1: Figure S1 and Additional file 2: Figure S2). For CDC-FC+, PP/IVIG were administered 3–4 times 1 week before surgery. Negative conversion upon FC was considered an indicator of successful desensitization. However, rituximab usually results in false-positive B-cell crossmatch. Therefore, decisions regarding successful desensitization were primarily based on the outcomes of the SAA, especially for patients with class II DSA.

For CDC + FC+, a 2–3-week desensitization schedule was designed on the basis of the initial CDC titer and DSA titer. Rituximab (375 mg/m^2^) and PP/IVIG were administered 3 or 4 times a week, and the immunologic status was re-assessed via repeated CDC and SAA, with appropriate modifications in the desensitization schedule. The targets were T-cell CDC-negativity and an MFI < 5000 of immunodominant DSA during the initial period. However, we subsequently progressed to transplantation with a much higher MFI value of DSA if enough responsiveness to desensitization was confirmed through negative conversion of crossmatch or C1q binding activity. Since June 2015, bortezomib, a proteasome inhibitor, has been administered to CDC + FC+ patients who did not respond to rituximab and 3–4 doses of PP/IVIG. Four doses of bortezomib were administered (1.3 mg/m^2^ per dose) for 2 weeks during desensitization. Usually, total 6–10 cycles of PP with IVIG (100 mg/kg per cycle) were performed for CDC + FC+ patients before KT.

Instead of basiliximab which is used for induction in negative-crossmatch KT, we routinely used anti-thymocyte globulin (ATG; 1.5 mg/kg) for 1–4 days after KT for positive-crossmatch KTs. Alike negative-crossmatch KT, tacrolimus-based triple immunosuppressive drugs were used for maintenance (tacrolimus, mycophenolate mofetil [MMF] and steroid), and the target trough levels of tacrolimus were 5–10 ng/ml during the first month after KT, followed by 3–7 ng/ml. MMF was initiated before transplantation at an initial dose of 1000–2000 mg/d. Steroid was administered from the day of transplantation at 500–1000 mg of intravenous methylprednisolone; thereafter, the dosage was tapered to that of oral prednisolone.

Using these protocols, we performed desensitization in 21 CDC + FC+ patients, but 4 were not able to undergo KT because of a sustained high titer of T-cell CDC or C1q positivity despite bortezomib administration. None of the CDC-FC+ patients failed to undergo KT.

### Analysis of clinical outcomes

The primary end points were death-censored graft survival and patient survival. Graft failure was defined as returning to dialysis or renal re-transplantation. Secondary end points were biopsy-proven rejection (BPAR), graft function and infectious complications. Renal biopsy was performed only when graft rejection was clinically suspected based on increased serum creatinine or proteinuria, in accordance with our hospital policy. BPAR was confirmed by transplant pathologists at our institution and antibody mediated rejection (ABMR) or T-cell mediated rejection (TCMR) was diagnosed in accordance with Banff 2007 classification [16]. Allograft function was assessed on the basis of eGFR calculated using the Chronic Kidney Disease Epidemiology formula [17]. For comparisons at each time point, we considered eGFRs of the patients with graft failure as zero regardless of their follow-up status to minimize bias.

We routinely screened for cytomegalovirus (CMV) viremia (CMV DNA > 1000 copies/ml) and BK virus (BKV) viremia (BKV DNA > 10,000 copies/ml) every 3–6 months for the first year and then annually among all patients. CMV was checked whenever the patient had fever. In case of positive-crossmatch KT, CMV was monitored every week for 1 month, then every 3–6 months unless CMV viremia was confirmed. No routine prophylaxis against CMV infections was administered but CMV was managed by preemptive strategy [18]. Prophylactic trimethoprim-sulfamethoxazole was administered for *Pneumocystis jirovecii* pneumonia (PJP) for a minimum of 6 months. For fungal prophylaxis, 4 ml nystatin was orally administered four times a day for 12 months. Urinary tract infection (UTI), pneumonia, bacteremia, and intraabdominal infection were considered only when pathogens were identified with correlated symptoms. Herpes zoster was diagnosed on the basis of typical skin lesions.

### Statistical analysis

Data of categorical variables were shown as numbers (frequencies), and chi square test or Fisher’s exact test were used when appropriate. For the comparison of continuous variables, the Kruskal–Wallis test was used, and data were expressed as median (interquartile range [IQR]). Post hoc analysis was conducted with Bonferroni’s method for the inter-group comparison of eGFR. To confirm the independent association between crossmatch positivity and eGFR, linear regression was applied for confounders. Survival outcomes were compared by Kaplan–Meier survival curves with log-rank tests and also adjusted using cox regression analysis. All analyses were performed using a standard software (SPSS v23.0; IBM, Armonk, NY, USA), and *P* < 0.05 was considered statistically significant.

## Results

### Baseline characteristics of patients

Among 330 LDKT patients, 274 were CDC-FC-, 39 were CDC-FC+, and 17 were CDC + FC+. As shown in Table 1, age of recipients and donors was similar in all groups. Recipients in the CDC-FC+ and CDC + FC+ groups displayed female predominance compared to those in the control group and vice versa with respect to donor sex. The duration of pretransplant dialysis was similar; however, it was greater in cases of re-transplantation in CDC-FC+ and CDC + FC+ groups than in the control group. Pretransplant diabetes and cardiovascular disease, which defined as myocardial infarction, coronary artery revascularization and stroke, showed similar incidence among the three groups.

**Table 1:** Baseline characteristics

Variables	CDC − FCM− (*n* = 274)	CDC − FCM+ (*n* = 39)	CDC + FC+ (*n* = 17)	*P*
Age, years	48 (36–55)	52 (45–57)	46 (41–56)	0.059
Sex, males	178 (65.0%)	6 (15.4%)	4 (2.1%)	< 0.001
Donor age, years	45 (34–52)	43 (33–53)	41 (29–49)	0.647
Donor sex, males	111 (40.5%)	24 (61.5%)	5 (29.4%)	0.004
Dialysis duration				0.228
Preemptive	95 (34.7%)	11 (28.2%)	4 (23.5%)	
≤ 1 year	118 (43.1%)	13 (33.3%)	8 (47.1%)	
> 1 year	61 (22.3%)	15 (38.5%)	5 (29.4%)	
Retransplantation	12 (4.4%)	6 (15.4%)	5 (29.4%)	< 0.001
Pretransplant DM	73 (26.0%)	13 (33.3%)	2 (11.8%)	0.245
Pretransplant CVD	20 (7.3%)	3 (7.7%)	1 (5.9%)	0.971

*CDC* Complement dependent cytotoxicity, *FC* Flow cytometry

### Immunologic status

Table 2 shows the immunologic status before and after desensitization in positive-crossmatch groups. In the CDC-FC+ group, 53.9% patients were positive for B-cell FC, and 46.1% patients were positive for both T- and B-cell FC. The median MFI ratio was 3.6 (maximum 18.3) for T-cell FC and 8.0 (maximum 53.3) for B-cell FC. Before desensitization, there was a median of 2 DSA specificities per patient (IQR, 1–3), and the most frequent type of immunodominant DSA was anti-HLA DR (56.4%). Median MFI for immunodominant DSA was 4219 (IQR, 2357–10,000; maximum, 12,802). After desensitization with a median of three doses of PP/IVIG (IQR, 3–4), DSA was obliterated in 30.8% patients, and the median MFI for immune-dominant DSA declined to 1902 (IQR, 0–4294; maximum, 11,979).

**Table 2:** Immunologic details before and after desensitization

Variables	CDC − FC+ (*n* = 39)	CDC + FC+ (*n* = 17)
FC positivity, % (B / T and B)	53.9 / 46.1	–
FC MFI ratio		
T cell positive (*n* = 21), median (IQR)	3.6 (2.9–6.8) Max 18.3	–
B cell positive (*n* = 39), median (IQR)	8.0 (4.4–17.4) Max 53.3	–
CDC positivity, % (B / T and B)	–	64.7 / 35.3
CDC titer		
T cell (AHG phase), number (1:2 / 1:4 / 1:8 / 1:32)	–	2 / 1 / 2 / 1
B cell (Warm phase), number (1:1 / 1:2 / 1:4 / 1:32)	–	3 / 6 / 7 / 1
Number of Pretransplant PP + IVIG, median (IQR)	3 (3–4) Max 8	6 (4–7) Max 11
Pre-desensitization DSA		
HLA Class, % (I / II / I + II)	17.9 / 53.8 / 28.3	17.6 / 35.3 / 47.1
Number, median (IQR)	2 (1–3)	5 (5–6)
Immunodominant DSA, % (A / B / DR / DQ)	12.8 / 15.4 / 56.4 / 15.4	11.9 / 17.6/ 52.9 / 17.6
Immunodominant MFI, median (IQR)	4219 (2357–10000) Max 12802	10951 (5732–14724) Max 18056
Sum of MFI, median (IQR)	6577 (3686–13580) Max 45735	14663 (7818–24202) Max 66434
Positive for C1q binding assay	Not examined	9 of 16, not examined in 1
Post-desensitization DSA		
HLA Class, % (None / I / II / I + II)	30.8 / 12.8 / 46.2 / 10.3	11.8 / 23.5 / 41.2 / 23.5
Number, median (IQR)	1 (0–2)	2 (1–3)
Immunodominant DSA, % (None/A / B / DR / DQ)	30.8 / 7.7 / 10.3 / 41.0 / 10.2	11.8 / 11.8 / 11.8 / 52.8 / 11.8
Immunodominant MFI, median (IQR)	1902 (0–4294) Max 11979	4379 (1492–10457) Max 19235
Sum of MFI, median (IQR)	2685 (0–5811) Max 32811	5250 (2264–15,844) Max 36252
Positive for C1q binding assay	Not examined	0 of 16, not examined in 1

*ATG* Anti-thymocyte globulin, *CDC* Complement dependent cytotoxicity, *DSA* Donor-specific antibody, *FC* Flow cytometry, *HLA* Human leukocyte antigen, *IVIG* Intravenous immunoglobulin, *MFI* Median fluorescent intensity, *PP* Plasmapheresis

In the CDC + FC+ group, 64.7% patients were positive for B-cell CDC, and 35.3% patients were positive for both T- and B-cell CDC. The maximum titer of CDC positivity was 1:32 for both T- and B-cell crossmatches. There was a median of 5 DSA specificities per patient (IQR 5–6), and the most frequent type of immunodominant DSA was also anti-HLA DR (52.9%). The median MFI for immunodominant DSA was 10,951 (IQR, 5732–14,724; maximum, 18,056). After desensitization with a median of 6 doses and maximum of 11 doses of PP/IVIG, DSA was obliterated in 11.8% of recipients and the median MFI for immunodominant DSA decreased to 4379 (IQR, 1492–10,457; maximum, 19,235). We assessed the C1q-binding ability in 16 subsequent cases before and after desensitization. Nine patients presented C1q-binding DSA before desensitization. Two patients with sustained C1q positivity after 1 or 2 weeks of desensitization received 4 doses of bortezomib before transplantation. Eventually, 8 of 9 (88.8%) patients presented negative conversion of C1q binding ability, although DSA was still positive with considerable MFI value (minimum, 2259; maximum, 19,235).

### Graft and patient survival

During a median follow-up of 37 (IQR, 22–52) months, 12 cases of death-censored graft failure were noted. The causes of graft failure were acute ABMR (5 CDC-FC-, 1 CDC-FC+), chronic ABMR (2 CDC-FC-, 1 CDC-FC+, and 1 CDC + FC+), acute tubular injury (1 CDC-FC-), or non-compliance (1 CDC-FC-). Four patients died of acute cerebral infarction (*n* = 1), gastrointestinal bleeding (*n* = 2), or hematologic malignancy (*n* = 1).

**Fig. 2:**
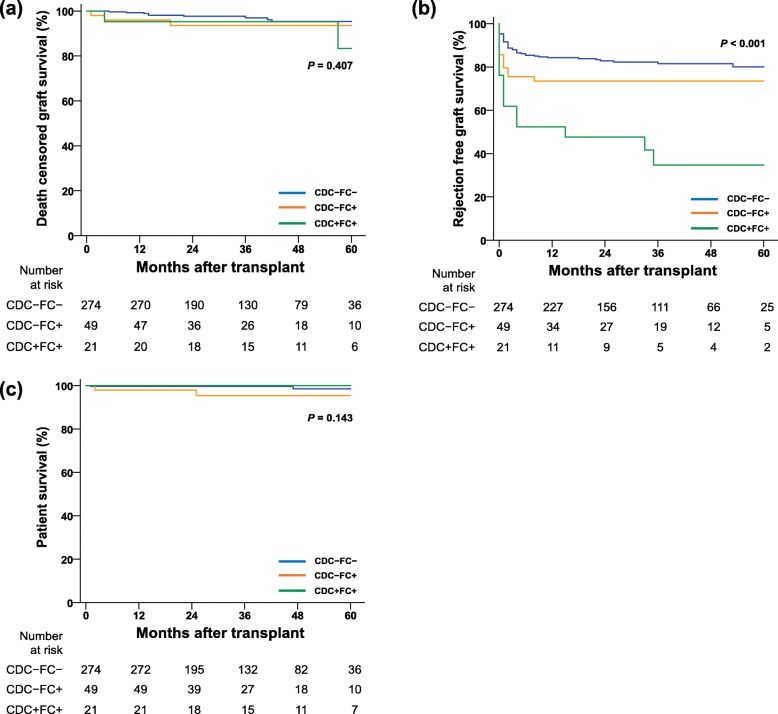
Comparison of (a) death censored graft survival, (b) rejection free survival, and (c) patient survival. CDC, complement dependent cytotoxicity; FC, flow cytometry.

On Kaplan Meier estimation of 5-year survival (Fig. 2), death-censored graft survival rates were 95.3% vs. 93.6% vs. 85.3% for CDC-FC-, CDC-FC+, and CDC + FC+, respectively, with no significant difference among them (*P* = 0.598). However, rejection-free graft survival was significantly lower in the CDC + FC+ group than in the other two groups (80.1% vs. 73.5% vs. 34.7% for CDC-FC-, CDC-FC+, and CDC + FC+, respectively; *P* < 0.001). Patient survival did not differ significantly among the three groups (*P* = 0.524), although events were too small to draw statistical conclusion.

There was no significant risk factor for death-censored graft survival in Cox analysis so we adjusted it with covariates which showed difference in demographics among three groups such as age, sex, donor sex, retransplantation. Patient survival and rejection-free survival was adjusted with factors of which *P* value was under 0.10 in univariate analysis; age and donor age for patient survival, donor age and dialysis duration for rejection-free graft survival. After adjustment, crossmatch positivity was still not associated with death-censored graft survival and patient survival. However, CDC + FC+ versus CDC-FC- (HR 5.29, 95% CI 2.84–987; *P* < 0.001) was independent risk factor for rejection-free graft survival after adjustment. Full results of Cox analysis are provided by Additional file 3: Table S1-S3.

### Graft function

**Fig. 3:**
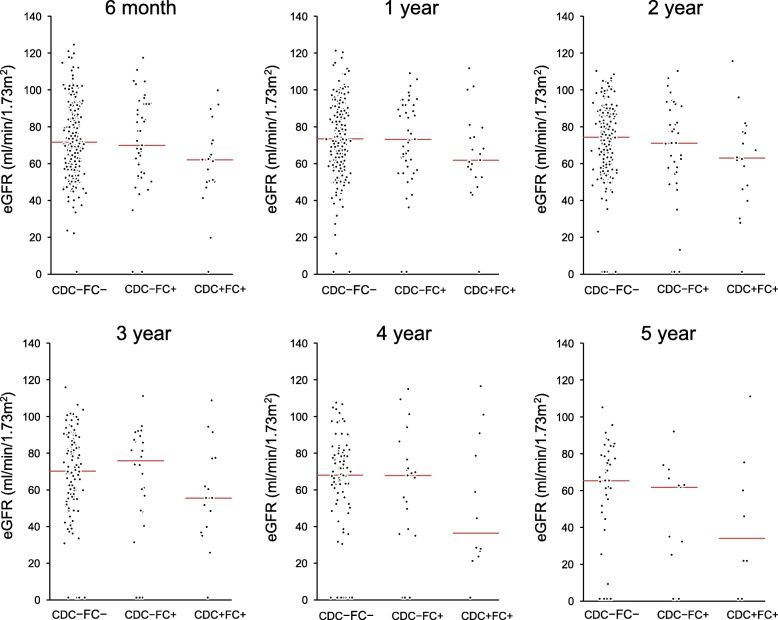
Graft function at each time point during 5 years of follow up. Red line means median value of eGFR. When graft failure occurred, eGFR was counted as zero. The differences in median eGFR were only significant at 6 months (*P* = 0.037), 1 year (*P* = 0.038), and 3 years (0.050) between CDC-FC- and CDC + FC+. CDC, complement dependent cytotoxicity; eGFR, estimated glomerular filtration rate; FC, flow cytometry

Figure 3 shows the distribution of eGFR during 5 years of follow-up. The CDC-FC- and CDC-FC+ groups showed a median eGFR of > 60 ml/min/1.73 m^2^ throughout the study period, although it slightly decreased with time. In the CDC + FC+ group, however, median eGFR declined to < 60 at 3 years and < 40 from the fourth year after KT. Furthermore, eGFRs of the CDC + FC+ group were lower than those of the other two groups throughout the follow-up period, although they differed significantly between the CDC-FC- and CDC + FC+ groups at 6 months (*P* = 0.037), 2 years (*P* = 0.038), and 3 years (*p* = 0.050). After adjustment with significantly associated factors in univariate analysis (sex, donor age and donor sex for eGFR at 6 months and 2 years; donor age for eGFR at 3 years), CDC + FC+ versus CDC-FC- was independently associated with eGFR at 6 months (β = − 8.18, *P* = < 0.001) and that at 2 years (β = − 5.85, *P* = 0.020).

### Biopsy-proven acute rejection within 1 year after transplantation

During posttransplant year 1, BPAR occurred in 42 CDC-FC- (15.3%) patients, 11 CDC-FC+ (28.2%) patients, and 8 CDC + FC+ (47.0%) patients. Although the number of rejections of crossmatch positive groups was too small to compare statistically, details regarding the first occurrence of BPAR and corresponding rescue treatment are compared in Table 3. The median duration from KT to BPAR was significantly shorter among CDC-FC+ (9 days; IQR, 5–31) and CDC + FC+ (14 days; IQR, 5–38) patients than in CDC-FC- patients (42 days; IQR, 11–109; *P* = 0.035). Among BPARs, ABMR was 42.9% and TCMR was 57.1% in CDC-FC- patients, while most types of rejections were ABMR in CDC-FC+ (90.9%) and CDC + FC+ (100%). Over half of the cases of rejection in the CDC-FC- group received only a steroid pulse or additional ATG as rescue treatment, while most patients in the CDC-FC+ (100%) and CDC + FC+ (75.0%) groups received PP/IVIG. Three (27.3%) patients in the CDC-FC+ and 5 (62.5%) in the CDC + FC+ group received PP/IVIG along with 4 doses of bortezomib upon DSA detection at high MFI or severe deterioration of allograft function.

**Table 3:** Rejection pathology and rescue treatments in the patients who experienced biopsy proven rejection within 1 year after transplantation

Variables	CDC − FC− (*n* = 42)	CDC − FC+ (*n* = 11)	CDC + FC+ (*n* = 8)	*P*
Median time to BPAR (days)	42 (11–109)	9 (5–31)	14 (5–38)	0.035
Type of acute rejection				< 0.001
ABMR	18 (42.9%)	10 (90.9%)	8 (100%)	
TCMR	24 (57.1%)	1 (9.1%)	0 (0%)	
Rescue treatment				< 0.001
Steroid pulse only	16 (38.1%)	0	2 (25.0%)	
Steroid pulse + ATG	7 (16.7%)	0	0	
PP/IVIG	19 (45.2%)	8 (72.7%)	1 (12.5%)	
PP/IVIG + bortezomib	0	3 (27.3%)	5 (62.5%)	

*ABMR* Antibody-mediated graft rejection, *ATG* Anti-thymocyte globulin, *BPAR* Biopsy-proven rejection, *CDC* Complement dependent cytotoxicity, *FC* Flow cytometry, *IVIG* Intravenous immunoglobulin, *PP* Plasmapheresis, *TCMR* T-cell medicated rejection

### Infectious complications within 1 year after transplantation

As shown in Table 4, the occurrence rate of urinary tract infection (7.7% vs. 51.3% vs. 23.5%, *P* < 0.001) was significantly higher in CDC-FC+ and CDC + FC+ groups than in the controls. Bacteremia (2.6% vs. 12.8% vs. 0%) and intraabdominal infection (0.7% vs. 7.7% vs. 0%, *P* = 0.036) were significantly more frequent in CDC-FC+ patients than in the other groups. CDC + FC+ patients experienced the highest rate of herpes zoster, PJP, and CMV infections, and CDC − FC+ patents also experienced more PJP and CMV infections than the controls (7.3% vs. 7.7% vs. 29.4%, *P* = 0.046 for herpes zoster; 0% vs. 2.6% vs. 11.8%, *P* = 0.001 for PJP; 13.9% vs. 48.7% vs. 64.7%, *P* < 0.001 for CMV infection; all the rates above were for CDC − FC− vs. CDC − FC+ vs. CDC + FC+, respectively). No patient died from posttransplant infection within 1 year.

**Table 4:** Infectious complication within 1 year after transplantation

Variables	CDC − FC− (*n* = 274)	CDC − FC+ (*n* = 39)	CDC + FC+ (*n* = 17)	*P*
UTI	21 (7.7%)	19 (51.3%)	4 (23.5%)	< 0.001
Bacterial pneumonia	4 (1.5%)	2 (5.1%)	0	0.234
Bacteremia	7 (2.6%)	5 (12.8%)	0	0.004
Intraabdominal infection	2 (0.7%)	3 (7.7%)	0	0.003
BKV viremia	18 (6.6%)	2 (5.1%)	0	0.527
Herpes zoster	20 (7.3%)	3 (7.7%)	5 (29.4%)	0.006
PJP	0	1 (2.6%)	2 (11.8%)	< 0.001
CMV viremia	38 (13.9%)	19 (48.7%)	11 (64.7%)	< 0.001

*BKV* BK virus, *CDC* Complement dependent cytotoxicity, *CMV* Cytomegalovirus, *FC* Flow cytometry, *PCP* Pneumocystis jirovecii pneumonia, *UTI* Urinary tract infection

## Discussion

Although patients undergoing crossmatch-positive KT have the advantage of increased survival compared with waitlist patients on dialysis, most studies reported that the outcome was inferior to that of compatible KT [2–4, 19]. In particular, the CDC + FC+ group showed poor graft survival, which has led to only few institutions performing this procedure. Our institution has actively performed CDC + FC+ KT and CDC-FC+ KT and achieved graft survival comparable to that of compatible KT with more advanced DSA-targeted desensitization compared to that in the present study. However, higher graft rejection, followed by lower graft function, especially in CDC + FC+, still warrants attention.

Our desensitization protocol was based on comprehensive interpretation of solid phase assays such as SAA and C1q assay as well as cell-based crossmatch. Marfo et al. [20] suggested a cutoff for transplantable MFI of DSA as < 5000 in positive-crossmatch patients who underwent desensitization, which was also indicated at our center during the initial period. However, we successfully performed KT for patients with higher DSA levels after desensitization. Although rejection occurred more frequently, most events were controllable and only 3 positive-crossmatch patients (4.2%) lost their grafts within 1 year owing to rejection. Furthermore, our indication for desensitization has been extended beyond the generally accepted criteria, being as high as T- or B-cell CDC-positivity titer of 1:32 and five median DSA types with maximum MFI of > 15,000. Nevertheless, patient and graft survival of positive-crossmatch KT was almost parallel to that of compatible KT. This surprising improvement was achieved through our DSA-targeted desensitization protocol and rescue treatment for acute rejection, especially through a well-planned approach for patients at very high risk.

The C1q-binding antibody assay has been recently reported as an indicator of allograft survival and responsiveness to rejection treatment [7, 21–23]. From August 2013, we monitored the C1q assay in all CDC + FC+ conversion trials and designed more extensive desensitization methods for C1q-positive patients. Furthermore, we utilized C1q-negative conversion as the target for successful desensitization and then progressed to KT.

Bortezomib has been introduced to effectively treat acute rejection in KT recipients [24–26]. Although it is uncertain whether bortezomib affects transplantability alone [27], bortezomib-based desensitization reportedly reduced anti-HLA DSA and increased the rate of KT in positive-crossmatch recipients [28]. During the study period, two patients with T- and B-cell CDC-positivity and C1q-binding DSA at high MFI level received 4 doses of bortezomib before transplantation. Despite the higher strength of DSA than that in patients who underwent CDC + FC+ KT before, two patients receiving bortezomib not only displayed successful KT outcomes but also did not experience acute rejection within 1 year after KT. Recently, we have routinely administered bortezomib for CDC + FC+ KT, expecting increased transplantability and decreased posttransplant acute rejection.

Another strength of our study was the more detailed comparison of infectious complications than previous studies among different immunologic risk groups. Although aggressive immunosuppression is inevitable, limited information is available regarding infectious complications in positive-crossmatch KT. Two studies reported that viral infection was increased in positive-crossmatch KT patients; however, they did not contain a control group [29, 30]. A recent multicenter study reported that the rates of readmission owing to infectious complications in both HLA-incompatible group and control subjects were virtually the same [31]. Furthermore, Okuda et al. [14] reported that bacterial and viral infections were not different between the positive- and negative-crossmatch KT groups. However, our study shows that various infections such as UTIs, bacteremia, PJP, and CMV infections were significantly more prevalent in the positive-crossmatch group than in the compatible group. Despite this increased risk of infection, CDC + FC+ and CDC-FC+ groups showed excellent patient survival. This suggests that positive-crossmatch KT potentially increases complications, however, they are controllable and do not increase patient mortality.

Unlike other studies, we excluded patients who showed CDC-FC- but had pretranspant DSA. The reasons were; 1) relatively small number (*n* = 21), 2) recent meta-analysis demonstrating low level DSA detected by Luminex alone couldn’t affect short to medium posttransplant outcome [15]. So, we thought that including those patients would make the analysis complicated rather than resulted in better risk stratification according to HLA antibody strength.

The limitation of our study is its retrospective single-center design with small population and small number of events. Also, we reported only a 5 year-outcome at maximum; hence, our results cannot be extrapolated to long-term setting. Finally, protocol biopsy was not performed in our institution, so we couldn’t compare chronic pathological results such as chronic ABMR or chronic allograft nephropathy.

## Conclusions

This study showed that intermediate graft and patient survival after CDC-FC+ and CDC + FC+ KT were improved to levels abreast with those after compatible KT, using a recent DSA-targeted desensitization protocol. Higher rejection rates are still a demanding challenge; thus, improved and specialized desensitization with advanced immunosuppressive agents is needed.

## Supplementary information

**Additional file 1: Figure S1.** Desensitization protocol for CDC-FC+ KT

**Additional file 2: Figure S2.** Desensitization protocol for CDC + FC+ KT

**Additional file 3: Table S1.** Cox analysis for death-censored graft survival. **Table S2.** Cox analysis for patient survival. **Table S3.** Cox analysis for rejection-free graft survival.

## Data Availability

The datasets for this study are available from the corresponding author on the reasonable request.

## Abbreviations

ABMR: Antibody-mediated graft rejection
ATG: Anti-thymocyte globulin
BKV: BK virus
BPAR: Biopsy-proven rejection
CDC: Complement dependent cytotoxicity
CMV: Cytomegalovirus
DSA: Donor-specific antibody
FC: Flow cytometry
HLA: Human leukocyte antigen
IVIG: Intravenous immunoglobulin
KT: Kidney transplantation
LDKT: Living donor kidney transplantation
MFI: Median fluorescent intensity
PJP: *Pneumocystis jirovecii* pneumonia
PP: Plasmapheresis
SAA: Single antigen assay
UTI: Urinary tract infection
