# Supplementary figures and images for: Transplant outcomes in positive complement-dependent cytotoxicity- versus flow cytometry-crossmatch kidney transplant recipients after successful desensitization: a retrospective study
Source: BMC Nephrol. 2019 Dec 9;20:456. doi: 10.1186/s12882-019-1625-2 (PMC6902609; doi:10.1186/s12882-019-1625-2)

**PP/IVIG (100mg/kg)**

**ATG (1.5mg/kg)**

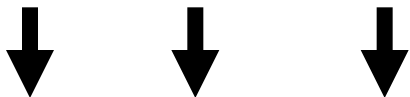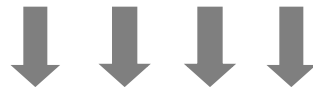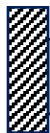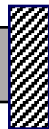

**-9**

**-7**

**-5**

**-2**

**-1**

**KT**

**+1**

**+2**

**+3**

**+4**

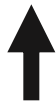

**RIT 375 mg/m<sup>2</sup>**

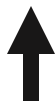

**CDC  
FC  
SAA**

**TAC+Steroid+MMF**

Supplement: Supplementary file 1 — Additional file 1: Figure S1. Desensitization protocol for CDC-FC+ KT [file 12882_2019_1625_MOESM1_ESM.pdf]

**PP/IVIG (100mg/kg) (6-10 times)**

**ATG (1.5mg/kg)**

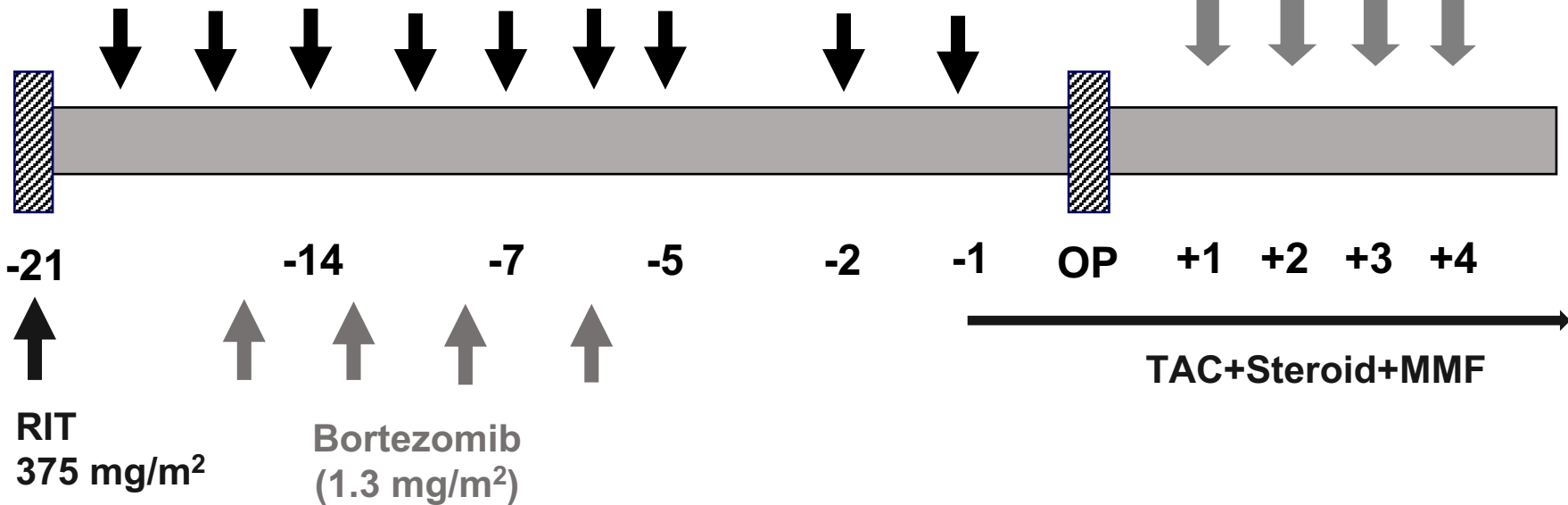

\* SAA, C1q: -14, -7, -2 day

\* CDC, FC: -2 day

Supplement: Supplementary file 2 — Additional file 2: Figure S2. Desensitization protocol for CDC + FC+ KT [file 12882_2019_1625_MOESM2_ESM.pdf]
