# Supplementary material for: Transplant outcomes in positive complement-dependent cytotoxicity- versus flow cytometry-crossmatch kidney transplant recipients after successful desensitization: a retrospective study
Source: BMC Nephrol. 2019 Dec 9;20:456. doi: 10.1186/s12882-019-1625-2 (PMC6902609; doi:10.1186/s12882-019-1625-2)
Supplement: Supplementary file 3 — Additional file 3: Table S1. Cox analysis for death-censored graft survival. Table S2. Cox analysis for patient survival. Table S3. Cox analysis for rejection-free graft survival. [file 12882_2019_1625_MOESM3_ESM.docx]

Table S1. Cox analysis for death-censored graft survival

|  | **Univariable Cox** |  |  | **Multivariable Cox^a^** | |
| --- | --- | --- | --- | --- | --- |
| **Variables** | **HR (95% CI)** | ***P*** |  | **HR (95% CI)** | ***P*** |
| Age, per 10yrs | 1.04 (0.98-1.10) | 0.183 |  | 1.04 (0.99-1.09) | 0.118 |
| Sex, male | 1.07 (0.37-3.07) | 0.906 |  | 1.31 (0.36-4.73) | 0.679 |
| Donor age, per 10yrs | 1.02 (0.97-1.08) | 0.423 |  |  |  |
| Donor sex, male | 1.50 (0.44-5.13) | 0.518 |  | 01.25 (0.41-3.78) | 0.693 |
| Dialysis duration, per 6 months | 0.99 (0.98-1.02) | 0.896 |  |  |  |
| Retransplantation | 1.29 (0.17-10.07) | 0.809 |  | 0.65 (0.08-5.34) | 0.666 |
| Pretransplant DM | 2.43 (0.74-7.97) | 0.143 |  |  |  |
| Pretransplant CVD | 3.00 (0.65-13.93) | 0.159 |  |  |  |
| CDC-FC- | Ref |  |  | Ref |  |
| CDC-FC+ | 1.82 (0.49-6.74) | 0.368 |  | 0.34 (0.06-1.87) | 0.215 |
| CDC+FC+ | 2.41 (0.52-11.24) | 0.263 |  | 0.69 (0.11-4.16) | 0.683 |

^a^ Multivariable Cox was done with covariates that were different among groups.

Table S2. Cox analysis for patient survival

|  | **Univariable Cox** |  |  | **Multivariable Cox^a^** | |
| --- | --- | --- | --- | --- | --- |
| **Variables** | **HR (95% CI)** | ***P*** |  | **HR (95% CI)** | ***P*** |
| Age, per 10yrs | 1.12 (0.99-1.26) | 0.058 |  | 1.25 (1.01-1.56) | 0.049 |
| Sex, male | 0.78 (0.11-5.54) | 0.804 |  |  |  |
| Donor age, per 10yrs | 1.31 (1.06-1.64) | 0.015 |  | 1.11 (0.96-1.29) | 0.166 |
| Donor sex, male | 0.85 (0.12-6.04) | 0.872 |  |  |  |
| Dialysis duration, per 6 months | 1.01 (0.98-1.03) | 0.486 |  |  |  |
| Retransplantation | 0.04 (0.01-103.10) | 0.719 |  |  |  |
| Pretransplant DM | 0.89 (0.09-8.58) | 0.922 |  |  |  |
| Pretransplant CVD | 3.69 (0.38-35.59) | 0.259 |  |  |  |
| CDC-FC- | Ref |  |  | Ref |  |
| CDC-FC+ | 5.12 (0.72-36.46) | 0.103 |  | 2.55 (0.32-20.30) | 0.377 |
| CDC+FC+ | cannot be calculated | 0.874 |  | cannot be calculated | 0.677 |

^a^ Multivariable Cox was done with covariates of which P <0.10 in univariate Cox.

Table S3. Cox analysis for rejection-free graft survival

|  | **Univariable Cox** |  |  | **Multivariable Cox^a^** | |
| --- | --- | --- | --- | --- | --- |
| **Variables** | **HR (95% CI)** | ***P*** |  | **HR (95% CI)** | ***P*** |
| Age, per 10yrs | 0.99 (0.98-1.02) | 0.753 |  |  |  |
| Sex, male | 0.90 (0.57-1.42) | 0.636 |  |  |  |
| Donor age, per 10yrs | 1.04 (1.02-1.06) | <0.001 |  | 1.04 (1.02-1.07) | <0.001 |
| Donor sex, male | 1.14 (0.72-1.81) | 0.570 |  |  |  |
| Dialysis duration, per 6 months | 0.99 (0.98-1.01) | 0.087 |  | 0.99 (0.98-1.02) | 0.104 |
| Retransplantation | 1.08 (0.47-2.50) | 0.851 |  |  |  |
| Pretransplant DM | 1.32 (0.81-2.14) | 0.267 |  |  |  |
| Pretransplant CVD | 1.05 (0.45-2.41) | 0.916 |  |  |  |
| CDC-FC- | Ref |  |  |  |  |
| CDC-FC+ | 1.61 (0.87-2.96) | 0.129 |  | 1.69 (0.92-3.13) | 0.093 |
| CDC+FC+ | 4.30 (2.33-7.93) | <0.001 |  | 5.29 (2.84-9.87) | <0.001 |

^a^ Multivariable Cox was done with covariates of which P <0.10 in univariate Cox.
